# Stathmin Mediates Hepatocyte Resistance to Death from Oxidative Stress by down Regulating JNK

**DOI:** 10.1371/journal.pone.0109750

**Published:** 2014-10-06

**Authors:** Enpeng Zhao, Muhammad Amir, Yu Lin, Mark J. Czaja

**Affiliations:** Department of Medicine and Marion Bessin Liver Research Center, Albert Einstein College of Medicine, Bronx, New York, United States of America; University of Windsor, Canada

## Abstract

Stathmin 1 performs a critical function in cell proliferation by regulating microtubule polymerization. This proliferative function is thought to explain the frequent overexpression of stathmin in human cancer and its correlation with a bad prognosis. Whether stathmin also functions in cell death pathways is unclear. Stathmin regulates microtubules in part by binding free tubulin, a process inhibited by stathmin phosphorylation from kinases including c-Jun N-terminal kinase (JNK). The involvement of JNK activation both in stathmin phosphorylation, and in hepatocellular resistance to oxidative stress, led to an examination of the role of stathmin/JNK crosstalk in oxidant-induced hepatocyte death. Oxidative stress from menadione-generated superoxide induced JNK-dependent stathmin phosphorylation at Ser-16, Ser-25 and Ser-38 in hepatocytes. A stathmin knockdown sensitized hepatocytes to both apoptotic and necrotic cell death from menadione without altering levels of oxidant generation. The absence of stathmin during oxidative stress led to JNK overactivation that was the mechanism of cell death as a concomitant knockdown of JNK1 or JNK2 blocked death. Hepatocyte death from JNK overactivation was mediated by the effects of JNK on mitochondria. Mitochondrial outer membrane permeabilization occurred in stathmin knockdown cells at low concentrations of menadione that triggered apoptosis, whereas mitochondrial β-oxidation and ATP homeostasis were compromised at higher, necrotic menadione concentrations. Stathmin therefore mediates hepatocyte resistance to death from oxidative stress by down regulating JNK and maintaining mitochondrial integrity. These findings demonstrate a new mechanism by which stathmin promotes cell survival and potentially tumor growth.

## Introduction

Stathmin 1 (STMN1) is a ubiquitous cytoplasmic protein that is a critical regulator of microtubules [1], [2]. Stathmin binds to unpolymerized tubulin and acts to destabilize microtubules by either sequestering free tubulin or promoting microtubule catastrophe [3], [4]. Stathmin regulates the microtubule dynamics of the mitotic spindle and therefore is most highly expressed in rapidly proliferating cells including many human cancers [3]. In the liver stathmin is expressed embryologically, lost after birth and re-expressed in hepatocytes and other cells in response to the regenerative stimulus of partial hepatectomy [5]–[7]. Hepatocellular carcinomas frequently express increased levels of stathmin that correlate with high tumor grade, vascular invasion and early recurrence [8]. Stathmin is also highly expressed in many other human cancers including breast, leukemia and prostate, and has been associated with poor histology, increased metastasis, increased drug resistance and decreased survival in these cancers as well [9]–[12].

Stathmin is regulated both transcriptionally and post-translationally. Central to stathmin regulation is its post-translational modification by phosphorylation at four serine residues (Ser-16, -25, -38 and -63) which acts to block stathmin association to free tubulin [3]. A number of kinases have been implicated in stathmin phosphorylation including cAMP-dependent protein kinase, cyclin-dependent kinases, and the mitogen-activated kinases (MAPK) extracellular signal-regulated kinase 1/2 (ERK1/2) and p38 [13]–[16]. Stathmin has also been identified as a substrate of the MAPK c-Jun N-terminal kinase (JNK) [17], [18], and stathmin expression is regulated transcriptionally by JNK-dependent c-Jun activation [19]. The fact that multiple kinases mediate stathmin phosphorylation suggests the importance of stathmin phosphorylation in cellular responses to a variety of stresses. However, the functional effects of phosphorylation are unclear as for example stathmin phosphorylation has been reported to both promote and inhibit cell death [17], [20].

JNK is a critical regulator of hepatocyte death resulting from a variety of forms of liver injury [21]–[23]. Among the forms of death regulated by JNK is that occurring from injurious levels of oxidative stress which is a common mechanism of hepatocyte death [24]. Studies in the menadione model of oxidant stress have demonstrated that RALA [21], [25], [26] and primary [27] hepatocytes are sensitized to death from menadione-induced oxidative stress in association with sustained overactivation of JNK/c-Jun signaling. In RALA hepatocytes, death from menadione is blocked by a genetic knockout of JNK1 [25], or the c-Jun dominant negative TAM67 [28], demonstrating that overactivation of JNK/c-Jun signaling mediates cell death from oxidant stress.

The known function of JNK in cellular resistance to hepatocyte death from oxidative stress, together with the fact that stathmin is a JNK substrate, led us to examine the role of stathmin in JNK-dependent hepatocyte death from oxidant stress. Menadione induced JNK-dependent stathmin phosphorylation. A stathmin knockdown sensitized cells to death from menadione in association with overactivation of JNK/c-Jun signaling. Death was JNK dependent as selective knockdown of JNK1 or JNK2 in cells lacking stathmin blocked death. These findings demonstrate a mutual regulation between stathmin and JNK that mediates cellular resistance to death from oxidative stress, and may impart a survival advantage from stathmin overexpression that occurs in human hepatocellular carcinoma.

## Materials and Methods

### Cell culture and treatments

Studies were performed in the rat hepatocyte line RALA255-10G (RALA hepatocytes) which is conditionally immortalized with a mutant SV40 virus expressing a temperature sensitive T antigen (kindly provided by Janice Y. Chou, NIH) [29]. Cells were routinely cultured in Dulbecco’s modified Eagle’s medium (Mediatech, Manassas, VA), 4% fetal bovine serum (Atlanta Biologicals, Lawrenceville, GA) and antibiotics (Invitrogen, Carlsbad, CA) at the permissive temperature of 33°C. Unless otherwise noted, for experiments trypsinized cells were plated and cultured at 33°C for 24 h, and then cultured in Dulbecco’s modified Eagle’s medium, 2% fetal bovine serum, antibiotics and 1 µM dexamethasone (Sigma, St. Louis, MO) at the restrictive temperature of 37°C for 72 h, as previously described [30]. These culture conditions suppress T antigen expression, and the cells become nontransformed and differentiated [29]. Cells were then placed in serum-free medium containing antibiotics and dexamethasone for 18 h prior to the start of an experiment.

Cells were treated as indicated with 40 −70 µM menadione (Sigma), 10 µM SP600125 (BD Biosciences, San Diego, CA), actinomycin D 15 ng/ml (Sigma) followed 1 h later by 15 ng/ml TNF (R&D Systems, Minneapolis, MN) or 10 µM Q-VD-OPh (MP Biomedicals, Aurora, OH). SP600125 and Q-VD-OPh were given for 1 h prior to menadione administration. Control cells received an equivalent amount of DMSO vehicle alone for studies involving SP600125 or Q-VD-OPh. Oleic acid (Sigma) was conjugated to bovine serum albumin as previously described [31], and administered at same time as the menadione.

### Protein isolation and Western blotting

Cellular total protein and mitochondrial and cytosolic protein fractions, were isolated as previously described [26], [32]. Protein concentrations were determined by the Bio-Rad (Hercules, CA) protein assay according to the manufacturer’s instructions. Western blotting was performed, as previously described [33], except transfers were performed with a Bio-Rad Trans-Blot Turbo Transfer System. Nitrocellulose membranes were exposed to antibodies that recognize phospho-Ser-16-stathmin, phospho-Ser-38-stathmin, total JNK, phosphorylated c-Jun, phosphorylated and total ERK1/2, phosphorylated MAPK kinase 4, caspase 3 and 7, tubulin (Cell Signaling, Beverly, MA), phosphorylated JNK, total c-Jun (Santa Cruz Biotechnology, Santa Cruz, CA), total stathmin (EMD Millipore, Billerica, MA), phospho-Ser-25-stathmin (Abcam, Cambridge, MA), β-actin (Sigma), cytochrome c (BD Biosciences) and cytochrome oxidase (MitoSciences, Eugene, OR). Western blot signals were quantitated by a FluorChem densitometer (Alpha Innotech), and stathmin signals were normalized to that for β-actin.

### Lentivirus construction and infection

The shRNA nucleotide sequences for *Stmn1*, *Jnk* and *c-Jun* are shown in Table 1. The shRNAs to *Jnk*, *Jnk1* and *Jnk2* have been successfully employed previously [25]. Oligonucleotides were annealed and cloned into the BglII-XhoI site of pSUPER (Life Technologies, Grand Island, NY). The SmaI-XhoI fragments of the corresponding pSUPER plasmids, which included the H1 promoter-shRNA cassette, were subcloned into the EcoRV-XhoI sites of the lentiviral vector pCCL.sin.PPT.hPGK.GFPWpre [34].

**Table 1:** shRNA Sequences.

*Stmn1 #1*	5′- GGAGGAAATTCAGAAGAAA-3′
*Stmn1 #2*	5′- GCAGAAGAAAGACGCAAGT-3′
*Jnk*	5′-GCATGGGCTACAAGGAGAA-3′
*Jnk1*	5′-GGAATAGTGTGTGCAGCTT-3′
*Jnk2*	5′-GGAATTGTTTGTGCTGCTT-3′
*c-Jun*	5′-GCTGATTACTGTCTATAAT-3′

Lentiviral stocks were produced by Fugene 6 (Promega, Madison, WI)-mediated transfection of the modified transfer vectors and the packaging vectors pMDLg/pRRE, pRSV-Rev and pMD2.VSVG into HEK-293T cells. Supernatants harvested at 48 h were titered by plaque assay, and the virus was infected into RALA hepatocytes at a multiplicity of infection of 5. Infection efficiency was determined by the numbers of green fluorescent protein positive cells under fluorescence microscopy at 72 h which exceeded 98% for all constructs. All experiments were performed in polyclonal, stably-infected cells.

### 3-(4,5-Dimethylthiazol-2-yl)-2,5-diphenyltetrazolium bromide (MTT) assay

Menadione-induced cell death was quantified by MTT assay [35]. At 24 h after menadione treatment, the cell culture medium was replaced by an equal volume of a 1 mg/ml MTT solution, pH 7.4, in Dulbecco’s modified Eagle’s medium. After incubation at 37°C for 1 h, the MTT solution was removed, the formazan product solubilized with 1-propanol and the absorbance of this compound measured at 560 nm in a spectrophotometer. The percentage of cell death was determined by dividing the optical density of the treated group by the optical density for untreated, control cells, multiplying by 100, and subtracting that number from 100.

### Fluorescence microscopy

The steady-state levels of apoptotic and necrotic cells were determined by acridine orange and ethidium bromide costaining cells under fluorescence microscopy, as previously described [36]. Hepatocytes with condensed or fragmented nuclei and a shrunken cytoplasm by acridine orange staining were considered apoptotic, and necrotic cells were detected by positive staining with ethidium bromide. A minimum of 400 cells per dish were examined, and the numbers of apoptotic and necrotic cells expressed as a percentage of the total number of cells.

### Adenoviruses

Adenoviruses were grown in 293 cells, purified on cesium chloride gradients, tittered by plaque assay and infected into RALA hepatocytes at an multiplicity of infection of 20, as previously described [30], [35]. Cells were infected with the control adenovirus Ad5LacZ which expresses the β-galactosidase gene [37], a Bcl-2-expressing adenovirus [38] or an adenovirus that expresses catalase [39].

### Superoxide assay

Superoxide production was determined by lucigenin chemiluminescence. Cells were exposed to 1 mg/ml of lucigenin in Krebs-Ringer solution. Chemiluminescence was measured by microplate reader and normalized to cellular protein.

### ATP assay

Intracellular ATP concentrations were determined by colorimetric kit (BioVision, Mountain View, CA) using the manufacturer's instructions. Values were normalized to protein concentration and expressed relative to untreated control cells.

### β-oxidation assay

Rates of fatty acid β-oxidation were determined by a modification of the method of Hoppel *et al*. [40], as previously described [31]. Rates of β-oxidation were measured from the production of radioactive carbon dioxide from the oxidation of [^14^C]-oleate. Rates are expressed as counts per million normalized to total protein.

### Statistical analysis

Numerical results are reported as mean ± S.E. and derived from at least three independent experiments. Groups were compared by the Student's t-test with statistical significance defined as *P*<0.05 using Sigma Plot (Jandel Scientific, San Rafael, CA).

## Results

### Stathmin is regulated by oxidant stress

**Figure 1:**
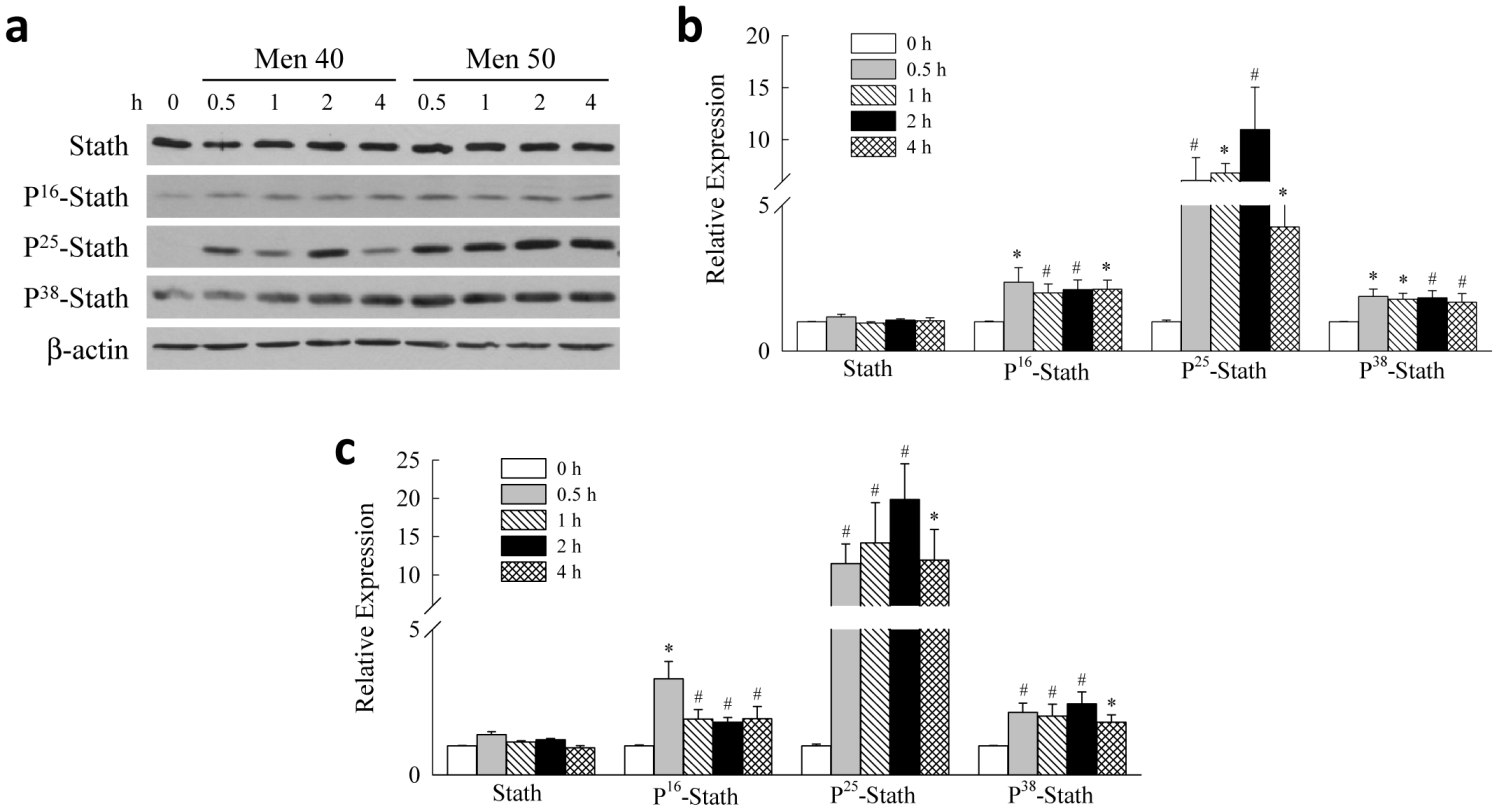
Menadione induces stathmin phosphorylation. (a) Wild-type RALA hepatocytes were treated with 40 or 50 µM menadione (Men) for the indicated times. Total cellular protein was isolated and immunoblotted for total stathmin (Stath), the indicated phospho-serine (P-) stathmin forms, and β-actin as a loading control. (b) Relative immunoblot band intensities for the indicated stathmin proteins normalized to the signal for β-actin as quantified by scanning densitometry in cells untreated or treated with 40 µM menadione for the indicated times (*P<0.02, ^#^ *P*<0.003 as compared to untreated cells; n = 5–7). (c) Densitometry scanning of immunoblots for cells treated with 50 µM menadione (*P<0.03, ^#^ *P*<0.005 as compared to untreated cells; n = 4–7).

To begin to delineate the function of stathmin in hepatocyte death from oxidant stress, we examined whether levels of total stathmin or its phosphorylated forms are altered in response to injurious oxidative stress. Stathmin expression was examined over time after treatment of RALA hepatocytes with the superoxide generator menadione at two concentrations that have been demonstrated to be nontoxic (40 µM) or to induce modest amounts of cell death (50 µM) [28]. Stathmin was constitutively expressed and levels of total stathmin determined by immunoblotting were unaffected with menadione treatment (Figure 1a). However, the levels of stathmin phosphorylated at Ser-16, Ser-25 and Ser-38 were all increased at both concentrations of menadione (Figure 1a). Densitometry scanning of band intensity confirmed that total stathmin protein levels were unaffected by menadione, whereas all three phosphorylated forms were significantly increased with both 40 µM (Figure 1b) and 50 µM (Figure 1c) menadione treatment. These findings suggest the possibility that post-translational modifications of stathmin modulate menadione toxicity.

**Figure 2:**
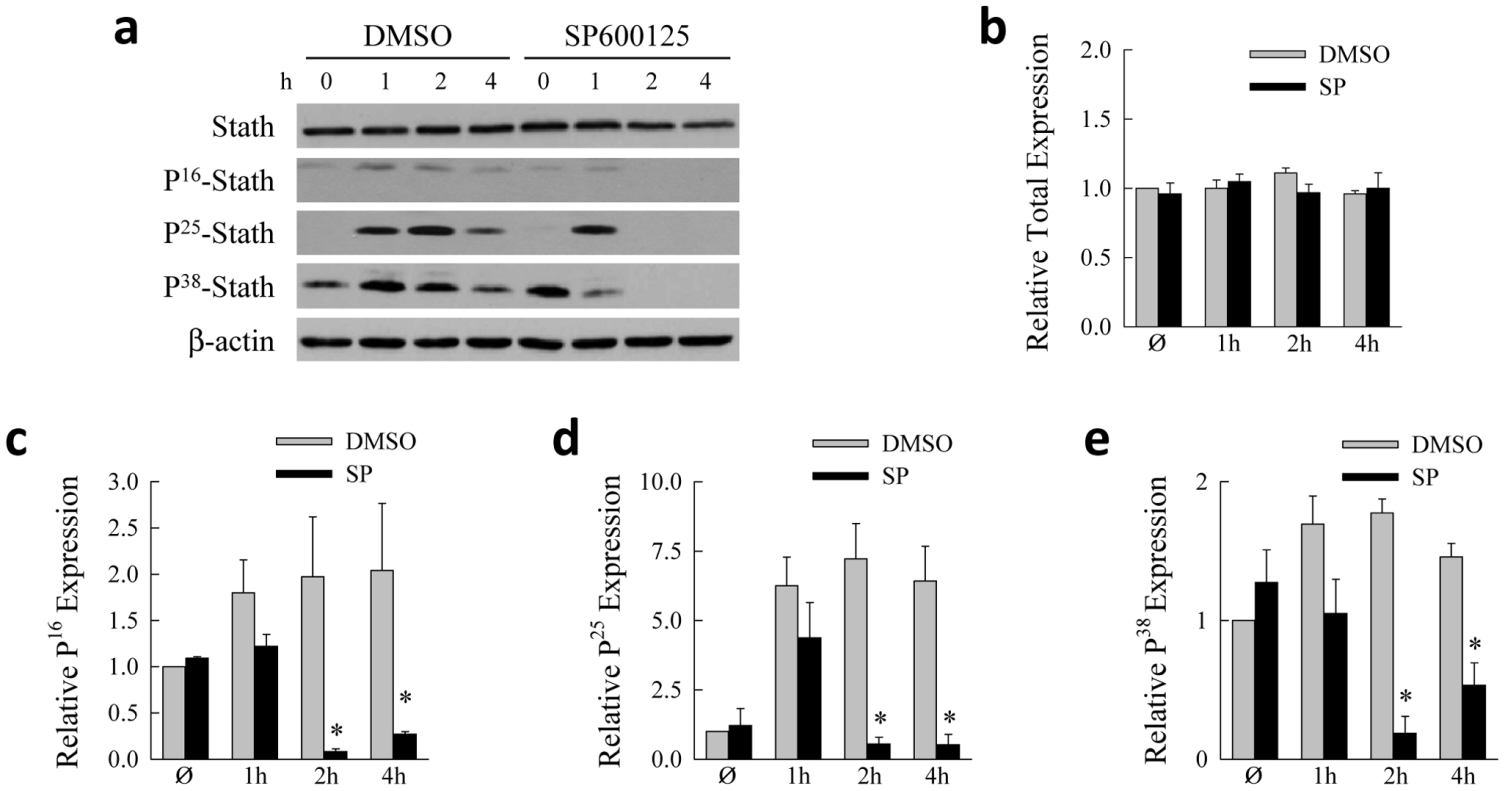
Menadione-induced stathmin phosphorylation is JNK dependent. (a) Wild-type cells were pretreated with dimethyl sulfoxide (DMSO) as vehicle control or SP600125, treated with 40 µM menadione for the indicated times, and their total protein isolated and immunoblotted with the antibodies shown. (b–e) Densitometric scanning of immunoblot band intensities for total stathmin (b), and stathmin phosphorylated at Ser-16 (c), Ser-25 (d) and Ser-38 (e) (*P<0.01, as compared to cells treated with DMSO; n = 3–6).

To determine whether the phosphorylation of stathmin in response to oxidant stress is JNK mediated, we examined the effect of the JNK inhibitor SP600125 on menadione-induced stathmin phosphorylation. As compared to cells treated with dimethyl sulfoxide (DMSO) vehicle, SP600125 decreased levels of Ser-16, Ser-25 and Ser-38 phosphorylation in response to menadione without affecting total stathmin expression (Figure 2a). Densitometric scanning results demonstrated a significant decrease in all three phosphorylated forms but not of total stathmin with JNK inhibition (Figures 2b–e). These results demonstrate that superoxide-induced oxidant stress induced stathmin phosphorylation in hepatocytes through a JNK-dependent mechanism.

### Knockdown of stathmin sensitizes RALA hepatocytes to menadione-induced apoptosis and necrosis

**Figure 3:**
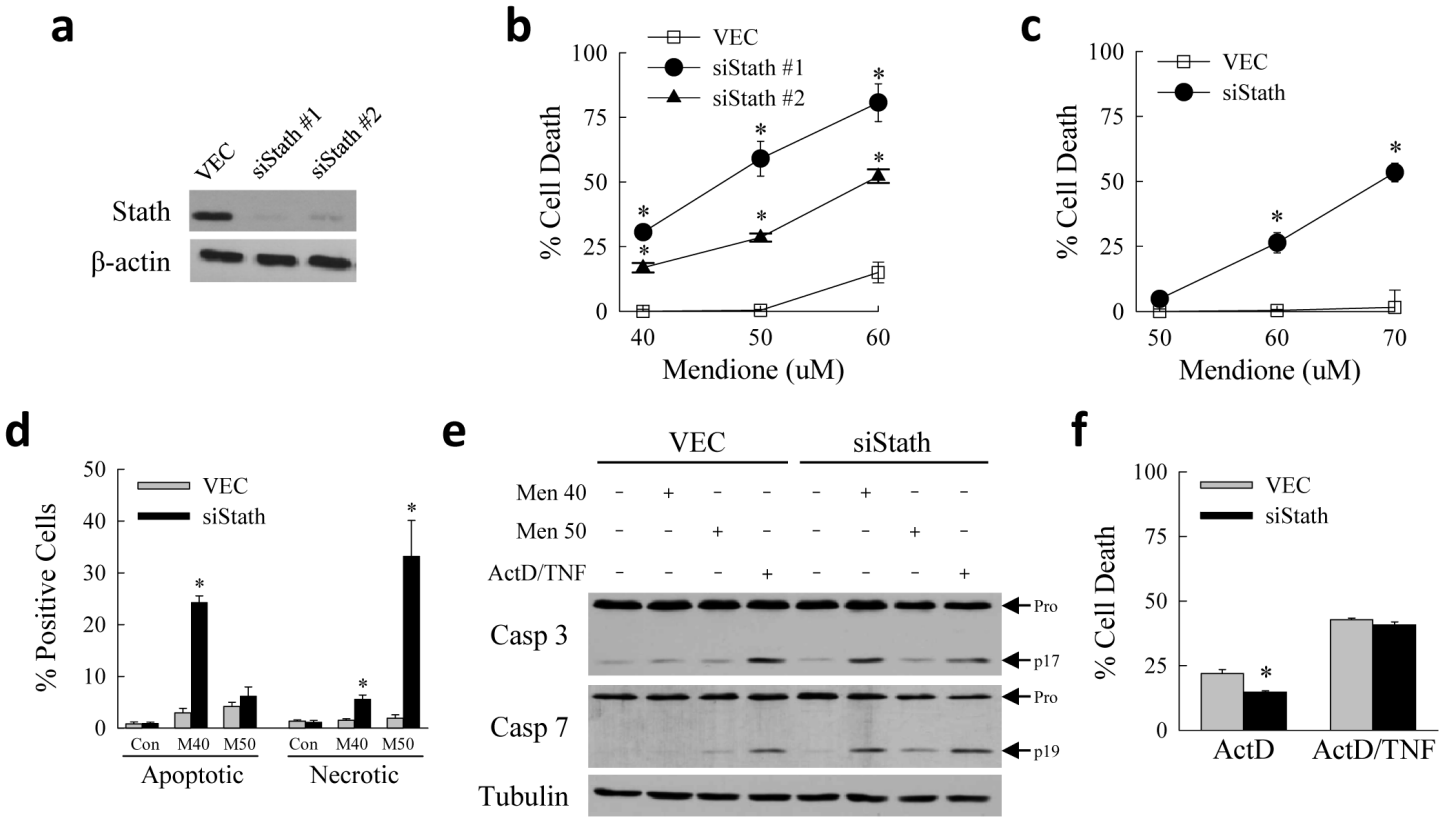
Stathmin blocks death from menadione-induced oxidant stress. (a) Total protein was isolated from RALA hepatocytes stably infected with a lentivirus containing vector alone (VEC), or expressing either of two shRNAs for stathmin (siStath #1 and #2), and immunoblotted with antibodies for stathmin and β-actin. (b) Percentage cell death by MTT assay in VEC and stathmin knockdown cells treated with the indicated concentrations of menadione for 24 h (*P<0.0001 as compared to VEC cells treated with the same concentration of menadione; n = 6). (c) Percentage cell death at 24 h in cells cultured under transformed conditions at 33°C and treated with the menadione concentrations shown (*P<0.0001 as compared to VEC cells treated with the same concentration of menadione; n = 5). (d) VEC and siStath cells were untreated or treated for 8 h with 40 (M40) or 50 (M50) µM menadione, costained with acridine orange/ethidium bromide, and the numbers of necrotic and apoptotic cells determined by fluorescence microscopy (*P<0.004 as compared to VEC cells treated with the same concentration of menadione; n = 4). (e) Total protein was isolated from VEC and siStath cells treated for 24 h with 40 or 50 µM menadione (Men) or actinomycin D+TNF (ActD/TNF) and immunoblotted with antibodies to caspase 3 or 7 and tubulin as a loading control. The procaspase (Pro) and cleaved caspase 3 (p17) and caspase 7 (p19) forms are indicated. (f) Percentage cell death by MTT assay in VEC and siStath cells after 24 h of treatment with actinomycin D (ActD) or actinomycin D+TNF (ActD/TNF) (*P<0.003 as compared to VEC cells with the same treatment; n = 4).

The increase in stathmin phosphorylation with oxidative stress suggested that this protein may modulate cellular resistance to oxidant-induced cell injury by either promoting or inhibiting cell death. To determine whether stathmin functions in oxidant-induced hepatocyte injury, RALA hepatocytes with a genetic knockdown of *Stmn1* were established. Two short hairpin RNAs (shRNAs) expressed by a lentiviral vector were identified that induced an effective knockdown of stathmin protein on immunoblots (Figure 3a). To determine the function of stathmin in oxidant stress, polyclonal RALA hepatocytes stably infected with a lentivirus containing vector alone (VEC cells) or expressing a *Stmn1* shRNA (siStath cells) were treated with increasing concentrations of menadione. The two independent knockdowns of stathmin sensitized the cells to death from low, normally nontoxic concentrations of 40–50 µM menadione, and increased levels of cell death from a higher, toxic concentration of 60 µM menadione, as determined by 24 h 3-(4,5-dimethylthiazol-2-yl)-2,5-diphenyltetrazolium bromide (MTT) assay (Figure 3b). Stathmin therefore mediates hepatocellular resistance to oxidant injury. siStath #1 cells (hereafter referred to as siStath cells) were employed for subsequent studies.

To additionally assess the function of stathmin in transformed cells, the effect of a stathmin knockdown on menadione-induced cell death was examined in RALA hepatocytes cultured at the permissive temperature of 33°C. Although RALA hepatocytes at 33°C were somewhat more resistant to death from menadione as expected of transformed cells with greater levels of antioxidants, knockdown of stathmin sensitized cells under these culture conditions to death from oxidant stress similar to findings in nontransformed RALA cells (Figure 3c). Thus, stathmin mediates hepatocyte resistance to oxidant injury in both transformed and nontransformed hepatocytes. These findings also indicate that the specific cell culture conditions required to maintain differentiated, nontransformed cells such as the inclusion of dexamethasone did not alter stathmin function in menadione-induced death.

To further confirm the increase in cell death in siStath cells cultured at the nontransformed temperature, and to determine the mode of death, the numbers of apoptotic and necrotic cells after menadione treatment were determined by fluorescence microscopy of acridine orange/ethidium bromide costained cells. With both 40 and 50 µM menadione, siStath cells had significantly increased steady-state numbers of apoptotic and/or necrotic cells (Figure 3d). At the lower 40 µM menadione concentration the levels of apoptosis and necrosis were significantly increased in knockdown cells but death was primarily apoptotic (Figure 3d). At the higher 50 µM menadione concentration, cell death in siStath cells was overwhelmingly necrotic (Figure 3d), reflecting the ability of higher levels of oxidant stress to convert apoptosis to death by necrosis [24], including that induced by menadione [41]. Consistent with the fluorescence microscopy findings of apoptosis, caspase 3 and 7 activation, as determined by the appearance of the cleaved, active forms of these proteins on immunoblots, increased in siStath cells with 40 µM menadione treatment (Figure 3e). Significant increases in caspase cleavage did not occur in siStath cells at the 50 µM menadione concentration (Figure 3e), in agreement with the fluorescence findings that death occurred from caspase-independent necrosis at this higher concentration. Significant caspase activation was not seen in VEC cells at either menadione concentration (Figure 3e).

To determine whether a generalized sensitization to caspase-dependent death had occurred in siStath cells, the effects of the stathmin knockdown on caspase-dependent apoptosis induced by the alternative death stimulus of actinomycin D and tumor necrosis factor (TNF) cotreatment was examined. Equivalent caspase activation occurred in both VEC and siStath cells from the apoptotic stimulus of actinomycin D/TNF cotreatment (Figure 3f). Consistent with equivalent actinomycin D/TNF-induced caspase activation in both cell types, death from actinomycin D/TNF was unaffected by stathmin knockdown and death from actinomycin D alone was even slightly decreased (Figure 3f). These data demonstrate that the protective effect of stathmin is specific for the death stimulus of oxidant stress as the stathmin knockout failed to sensitize cells to TNF death receptor-induced caspase activation.

**Figure 4:**
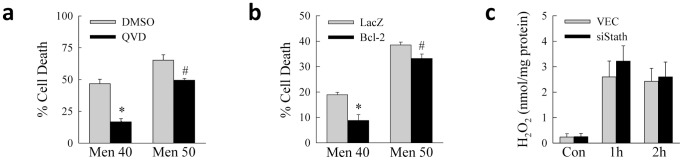
Cell death is blocked by caspase inhibition and Bcl-2. (a) Percentage cell death by MTT assay 24 h after treatment with vehicle dimethyl sulfoxide (DMSO) or the caspase inhibitor Q-VD-OPh (QVD) (*P<0.001, ^#^ *P*<0.01 as compared to VEC cells; n = 4). (b) Percentage cell death in siStath cells infected with the control adenovirus AdLacZ or a Bcl-2-expressing adenovirus and treated with 40 or 50 µM menadione for 24 h (*P<0.001, ^#^ *P*<0.02 as compared to VEC cells; n = 9–10). (c) Levels of hydrogen peroxide (H_2_O_2_) generated in VEC and siStath cells that were untreated controls (Con) or treated with 50 µM menadione for 1 or 2 h (n = 4–6).

To further demonstrate that loss of stathmin sensitizes to both apoptosis and necrosis from oxidant stress, we investigated the effect of caspase inhibition on cell death. The caspase inhibitor Q-VD-OPh markedly reduced cell death from 40 µM menadione and had a much smaller, albeit still significant, effect on death from 50 µM menadione (Figure 4a). Similarly adenoviral expression of the anti-apoptotic protein Bcl-2 also blocked death at both menadione concentrations, but to a greater extent at the lower, apoptotic dose (Figure 4b). In total the findings demonstrate that stathmin has an essential function in mediating resistance to both apoptotic and necrotic hepatocyte death from oxidative stress.

### Loss of stathmin does not increase superoxide production from menadione

A potential mechanism by which stathmin could modulate menadione toxicity is through an effect on the amount of menadione-generated reactive oxygen species. To examine this possibility, levels of menadione-induced superoxide were determined in control and knockdown cells. Previous studies demonstrated that superoxide generation peaks at 1 h after menadione administration in RALA hepatocytes [42]. Equivalent, low levels of superoxide were present in untreated VEC and siStath cells (Figure 4c). In response to menadione, superoxide levels increased markedly, but equally, at 1 and 2 h in VEC and siStath cells (Figure 4c), indicating that sensitization to menadione toxicity by stathmin knockdown was not due to increased superoxide production.

### Loss of stathmin leads to overactivation of JNK/c-Jun signaling

**Figure 5:**
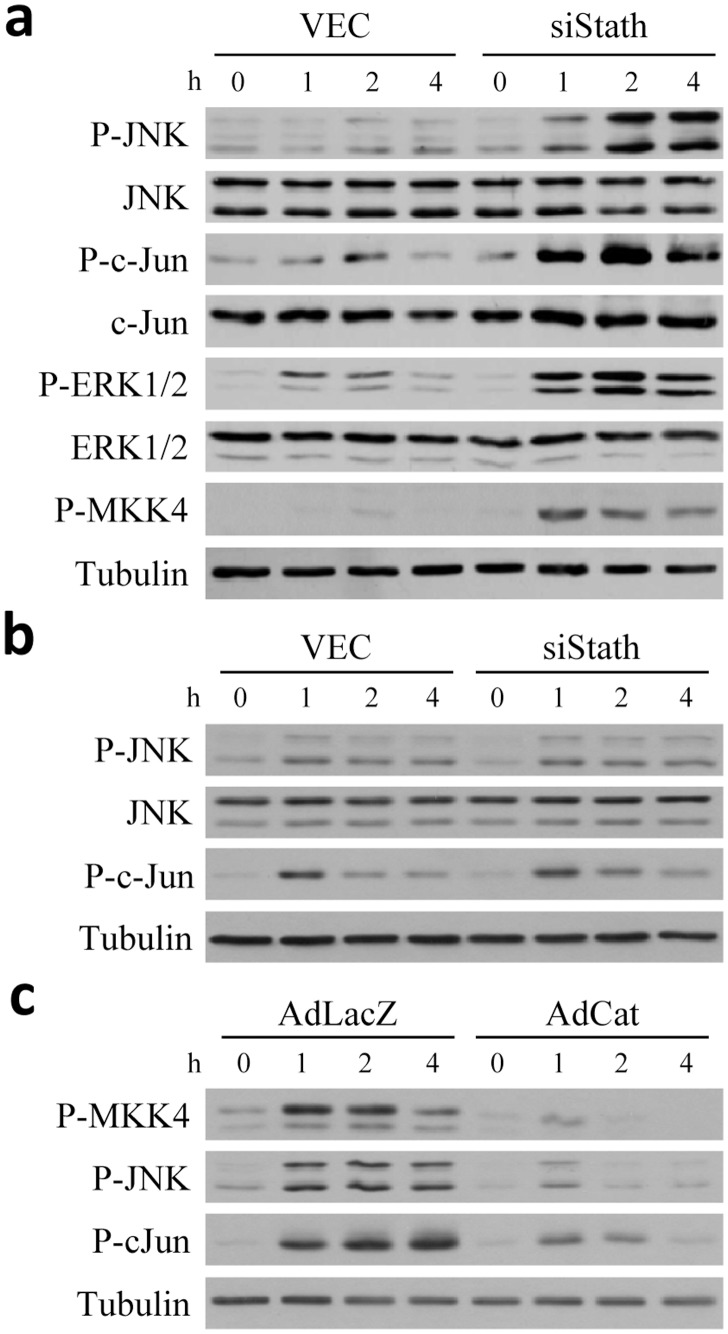
Loss of stathmin leads to JNK overactivation in response to oxidant stress. (a) RALA hepatocytes were treated with 40 µM menadione for the indicated number of hours. Total protein was isolated and immunoblotted for phospho- (P-JNK) and total (JNK) JNK, phospho- (P-c-Jun) and total (c-Jun) c-Jun, phospho- (P-ERK1/2) and total (ERK1/2) ERK1/2, phospho-MKK4 (P-MKK4) and tubulin. (b) Immunoblots of protein from cells treated with actinomycin D+TNF for the indicated number of hours. (c) siStath cells were infected with the control virus AdLacZ or the catalase-expressing virus AdCat, treated with 40 µM menadione for number of hours indicated and cells harvested for total protein isolation and immunoblotting with the antibodies shown.

Previous studies have demonstrated that RALA [21], [25], [26] and primary [27] hepatocytes are sensitized to death from menadione-induced oxidative stress by the mechanism of prolonged JNK/c-Jun activation. In RALA hepatocytes, this conclusion is supported by findings that death from menadione is blocked by a genetic knockout of JNK1 [25] or expression of the c-Jun dominant negative TAM67 [28]. Given this critical involvement of JNK signaling in hepatocyte death from oxidant stress, we examined whether loss of stathmin function affected JNK activation by menadione. In the absence of stathmin, increased and temporally sustained JNK and c-Jun activation occurred from menadione-induced oxidant stress as indicated by increased levels of phosphorylated JNK and c-Jun (Figure 5a). ERK1/2 overactivation also occurred in concert with increased JNK/c-Jun activation (Figure 5a), as we have previously reported [28]. In contrast, JNK activation in response to actinomycin D/TNF was unaffected by a stathmin knockdown (Figure 5b), consistent with the failure of the loss of stathmin to affect death from ActD/TNF (Figure 3f). JNK overactivation was secondary to upstream kinase activation as MAPK kinase 4 (MKK4) phosphorylation was also increased in knockdown cells (Figure 5a).

JNK/c-Jun activation in knockdown cells was dependent on oxidative stress generated by menadione. Adenoviral overexpression of the antioxidant enzyme catalase to block menadione-induced oxidant stress almost completely inhibited JNK and c-Jun phosphorylation from menadione treatment (Figure 5c). Although JNK/c-Jun overactivation could be secondary to the greater amount of cell death occurring in siStath cells, increased JNK/c-Jun phosphorylation was seen in knockdown cells within 1 h of menadione treatment, long before the 8 h onset of cell death. Thus, JNK/c-Jun overactivation is a primary effect of the stathmin knockdown, indicating that a negative feedback loop exists in which JNK-stimulated stathmin then functions to down regulate JNK.

### Increased JNK/c-Jun signaling mediates menadione toxicity from the loss of stathmin

**Figure 6:**
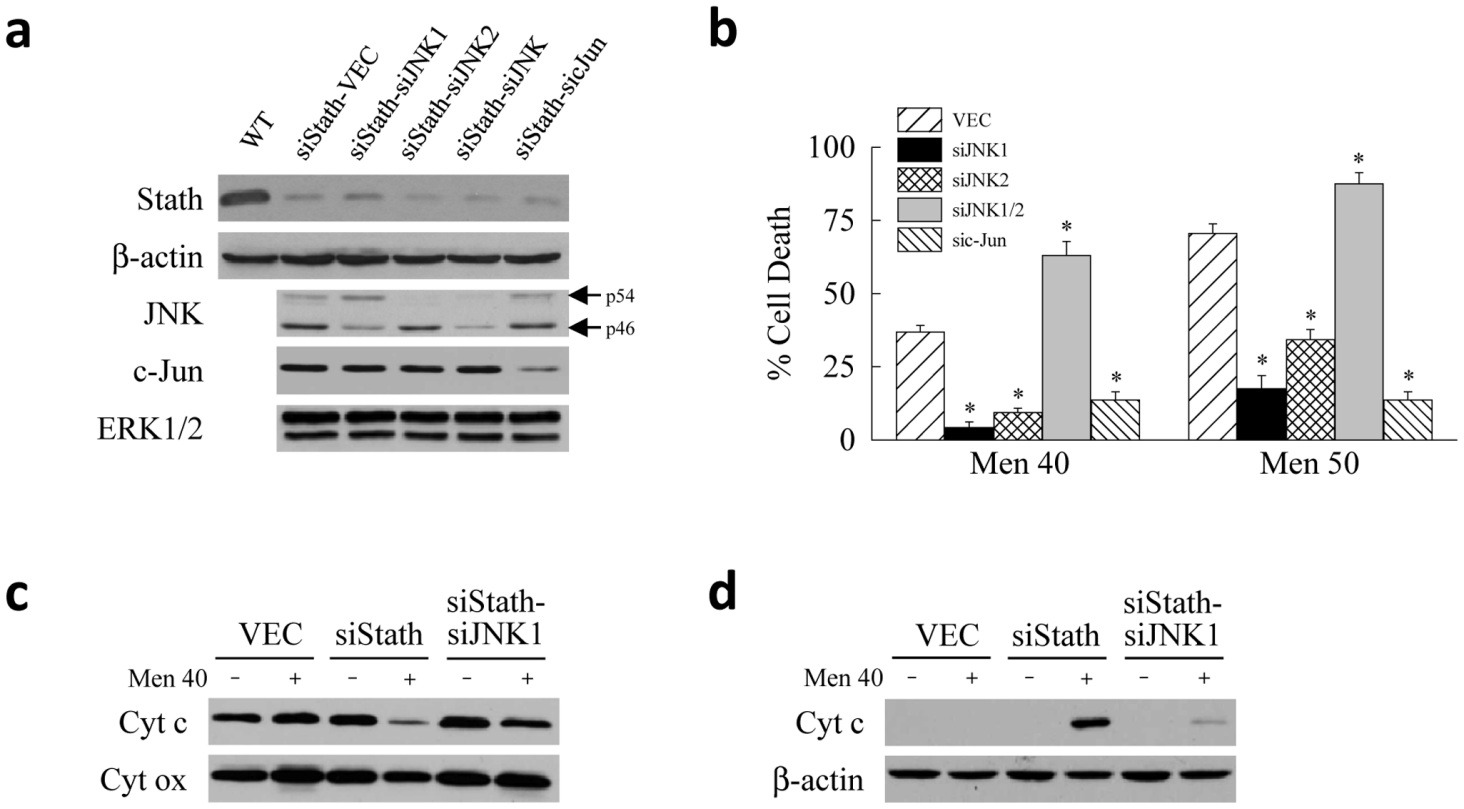
Overactivation of JNK/c-Jun signaling mediates cell death from menadione in stathmin knockdown cells in association with activation of the mitochondrial death pathway. (a) Immunoblots of total protein from wild-type (WT) and siStath cells with knockdowns of JNK and c-Jun for stathmin, β-actin, total JNK, c-Jun and ERK1/2. The p54 and p46 JNK forms are indicated. (b) Percentage cell death at 24 h by MTT assay in JNK and c-Jun knockdown cells after treatment with the indicated menadione concentrations (*P<0.001 as compared to VEC cells; n = 6). (c) Immunoblots of mitochondrial protein from VEC, siStath and siStath-siJNK1 cells untreated or treated with 40 µM menadione and probed for cytochrome c (Cyt c) and cytochrome oxidase (Cyt ox) as a loading control. (d) Cytosolic protein from the same cells immunoblotted for cytochrome c and β-actin.

Knockdown of stathmin promoted JNK/c-Jun overactivation suggesting that increased JNK/c-Jun signaling may be the mechanism sensitizing siStath cells to menadione killing. To examine this possibility, we established siStath cells with stable, lentiviral shRNA-mediated knockdowns of *Jnk1* (siStath-siJNK1 cells), *Jnk2* (siStath-siJNK2 cells), both *Jnk* genes (siStath-siJNK cells) and *c-Jun* (siStath-sicJun cells). Control cells were siStath cells secondarily infected with vector alone (siStath-VEC cells). As previously described [25], the *Jnk1* shRNA predominantly decreased levels of p46 JNK, the *Jnk2*-targeted shRNA reduced p54 JNK, and the shRNA directed against a common sequence of both genes reduced both protein forms (Figure 6a). The shRNA to *c-Jun* decreased c-Jun protein levels without affecting JNK (Figure 6a). ERK1/2 levels were unaffected by the *Jnk* and *c-Jun* knockdowns (Figure 6a). siStath-VEC and siStath-JNK/c-Jun knockdown cells were treated with menadione and the amount of death determined at 24 h by MTT assay. Knockdown of both JNK forms failed to protect against cell death and in fact significantly increased death (Figure 6b), consistent with our previous finding that pharmacological global JNK inhibition promotes cell death by blocking the beneficial cell proliferative effects of early, transient JNK activation [25]. In contrast, a selective knockdown of either JNK1 or JNK2 significantly decreased death from menadione in siStath cells, as did the knockdown of c-Jun (Figure 6b). These findings demonstrate that overactivation of JNK/c-Jun signaling is the mechanism of the increased sensitivity of stathmin-deficient cells to death from oxidant stress.

### Loss of stathmin increases death from menadione by compromising mitochondrial integrity

A central mechanism of hepatocyte death, including that from menadione-induced oxidant stress, is mitochondrial membrane permeabilization and dysfunction [43]. Cell injury can lead to permeabilization of the outer mitochondrial membrane and release of cytochrome c that triggers caspase 3 and 7 activation and apoptosis, or opening of the mitochondrial permeability transition pore in the inner membrane leading to membrane depolarization, cessation of ATP synthesis and necrosis. Our previous studies demonstrated that a critical determinant of hepatocyte resistance to oxidative stress from menadione is the maintenance of cellular ATP content [42], and that JNK signaling is essential to maintain rates of mitochondrial β-oxidation at levels that generate sufficient ATP for cell survival [25]. With findings that stathmin increases both death from oxidant stress and JNK activation, we examined menadione-treated cells for mitochondrial effects from a loss of stathmin.

**Figure 7:**
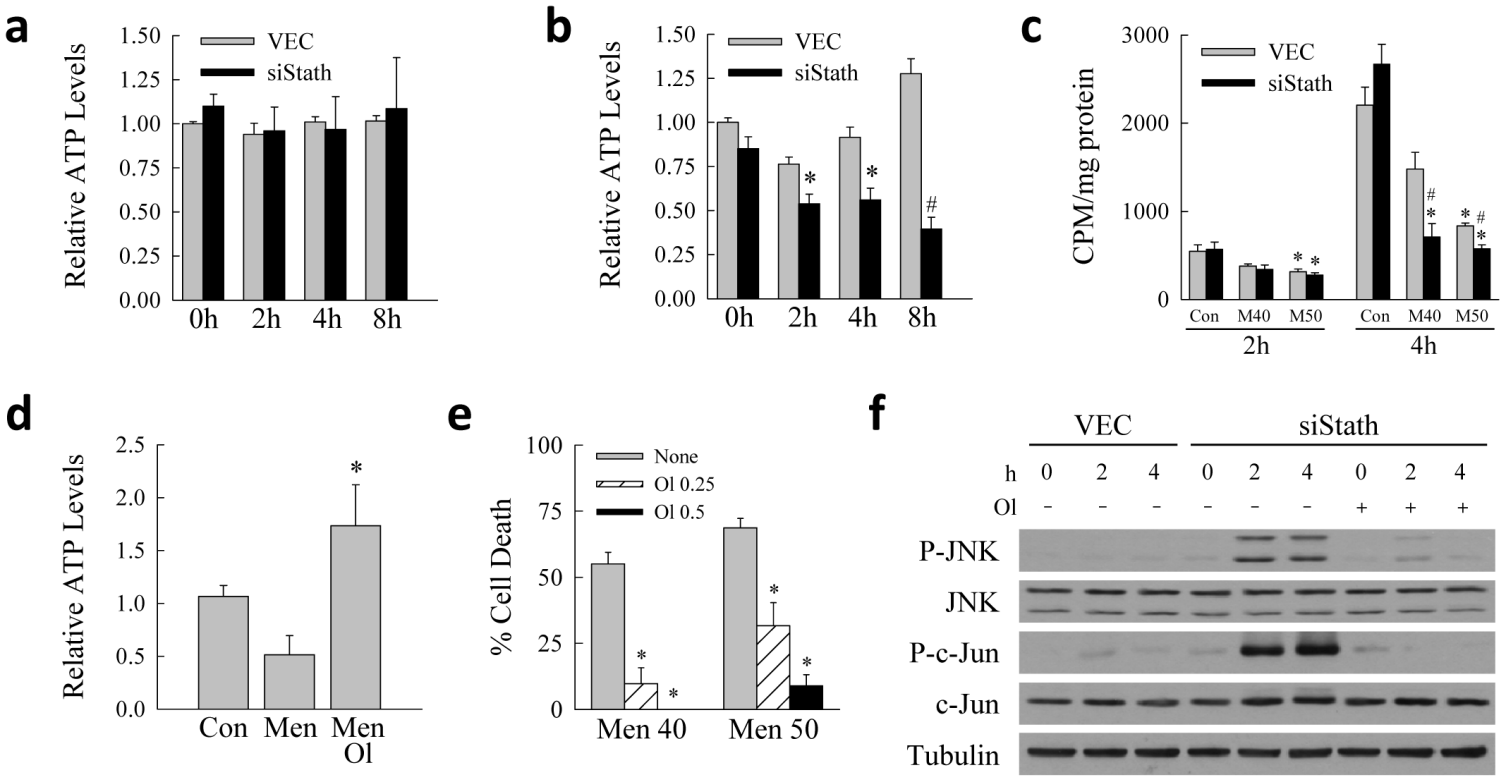
Loss of stathmin compromises mitochondrial function. (a) Relative ATP levels in VEC and siStath cells at the indicated times after treatment with 40 µM menadione (n = 4–5). (b) Relative ATP levels in VEC and siStath cells at the indicated times after treatment with 50 µM menadione (*P<0.01, ^#^ *P*<0.001 as compared to VEC cells with the same menadione treatment; n = 5–6). (c) Levels of β-oxidation measured as counts per million (cpm) per mg of cellular protein in VEC and siStath cells treated with menadione for 2 or 4 h (*P<0.03 as compared to untreated VEC cells; ^#^ *P*<0.03 as compared to VEC cells identically treated with menadione; n = 3). (d) Relative ATP levels in untreated control (Con), 8 h 50 µM menadione treated (Men) and menadione and 0.5 mM oleate cotreated (Men Ol) siStath cells (*P<0.002 as compared to menadione-treated cells without oleate; n = 4–6). (e) Percentage cell death by MTT assay in siStath cells 24 h after 40 or 50 µM menadione treatment alone (None) or with 0.25 or 0.5 mM oleate (Ol) cotreatment (*P<0.002 as compared to cells without oleate; n = 4–6). (f) VEC and siStath cells were treated with 40 µM menadione alone or cotreated with 0.5 mM oleate for the indicated hours. Total protein was isolated and immunoblotted for the proteins shown.

Outer mitochondrial membrane permeabilization occurred in siStath cells treated with 40 or 50 µM menadione as indicated by the decrease in mitochondrial levels of cytochrome c (Figure 6c and data not shown) and its presence in the cytosol (Figure 6d and data not shown) in knockdown but not control cells. Cytochrome c release was JNK-dependent as it was largely blocked in siStath cells with a JNK1 knockdown (Figures 6c and 6d). No significant change in cellular ATP content occurred in VEC or siStath cells after treatment with 40 µM menadione (Figure 7a). In contrast, at 50 µM menadione a significant decrease in ATP occurred in VEC cells within 2 h after menadione treatment with levels returning to normal by 4 h (Figure 7b). In siStath cells the decrease in ATP was significantly greater at 2 h and was sustained over 8 h (Figure 7b). Apoptotic menadione-induced death resulting from an absence of stathmin was therefore associated exclusively with outer membrane permeabilization, whereas the higher, necrosis-inducing concentration of menadione also led to compromise of ATP homeostasis.

Mitochondrial β-oxidation maintains ATP levels during menadione-induced oxidant stress [42], and decreased ATP at the higher menadione concentration suggested compromise of this metabolic pathway in knockdown cells. At 2 h after menadione treatment, levels of β-oxidation were decreased equally in control and knockdown cells only with 50 µM menadione (Figure 7c). After 4 h of menadione treatment the levels of β-oxidation were significantly decreased in siStath cells with both 40 and 50 µM menadione, but only at the higher concentration in VEC cells (Figure 7c). For both concentrations of menadione the decrease in β-oxidation was significantly greater in stathmin knockout cells (Figure 7c). Thus, in the absence of stathmin hepatocytes developed a more profound decrease in rates of mitochondrial β-oxidation and cellular ATP content.

To determine whether the decrease in ATP mediated death in stathmin knockout cells, the effect on cell death of supplementation with the free fatty acid oleate to increase β-oxidation rates and ATP content was examined. Oleate supplementation effectively reversed the menadione-induced decrease in ATP in siStath cells (Figure 7d). A concentration-dependent inhibition of cell death by oleate occurred at both the 40 and 50 µM menadione concentrations (Figure 7e), demonstrating that mitochondrial compromise and ATP depletion were a mechanism of death in stathmin-deficient cells. Interestingly, an effect was even seen at 40 µM menadione, a concentration at which ATP depletion was not detected. This effect may have resulted from the ability of oleate to block JNK activation as detected by the prevention by oleate of JNK and c-Jun phosphorylation in menadione-treated siStath cells (Figure 7f).

## Discussion

Stathmin is phosphorylated during mitosis and this post-translational modification has been mechanistically linked to cell proliferation. In addition, injurious stress from hyperosmotic shock and TNF has been demonstrated to increase stathmin phosphorylation [17], [20]. Although oxidative stress from hydrogen peroxide has been previously reported not to alter stathmin phosphorylation [17], the present study demonstrates that superoxide-mediated oxidant stress induces stathmin phosphorylation at serine-16, -25 and -38 in hepatocytes. In contrast to findings with hyperosmotic stress and TNF in which knockdowns of stathmin reduced cell death [17], [20], stathmin knockdown in hepatocytes under oxidative stress demonstrated that stathmin has a critical function in hepatocyte resistance to death from superoxide. These findings therefore delineate a new survival function for post-transcriptionally modified stathmin in cells under oxidant stress.

Stathmin protects against hepatocyte death from oxidant stress by a novel mechanism that is independent of this protein’s main function of regulation of microtubule stability. Stathmin down regulated JNK/c-Jun signaling in hepatocytes which promoted cell survival by preventing the deleterious effects of sustained JNK activation on mitochondria. Overactivation of JNK/c-Jun is the central mechanism of a variety of forms of liver injury [23]. Whereas transient JNK activation has beneficial cellular effects such as the promotion of proliferation, sustained activation has been demonstrated to sensitize hepatocytes to death from oxidant stress [27], [44], TNF [32], [45], [46], and acetaminophen [47]. How prolonged JNK activation promotes cell death remains unclear. However, evidence to date has implicated JNK compromise of mitochondrial integrity. Reported effects of sustained JNK signaling on hepatocytes have included mitochondrial outer membrane permeabilization [32] and impairment in energy homeostasis [25], [48]. Stathmin prevented both the release of apoptogenic mitochondrial proteins such as cytochrome c and ATP depletion that compromised cellular energy homeostasis and led to necrosis. These findings are consistent with the concept that apoptosis and necrosis are part of a continuum of cell death that shifts to necrosis in the absence of ATP. Stathmin has a central function in maintaining mitochondrial integrity during oxidant stress by down regulating JNK to prevent both forms of cell death. Interestingly the effect was specific for the death stimulus of oxidant stress as TNF toxicity was unaffected by a loss of stathmin. The reason for this specificity is that TNF, in contrast to menadione, failed to alter hepatocyte stathmin expression or phosphorylation and as a result loss of stathmin did not affect TNF-induced JNK activity (data not shown). These findings are in contrast to those in L929 cells in which TNF induced stathmin phosphorylation that promoted cell death [20], again emphasizing the cell type specific nature of stathmin phosphorylation and function.

Free tubulin can bind to and close the voltage-dependent anion channel of the mitochondrial outer membrane to regulate mitochondrial membrane permeability and metabolic function [49]–[51]. Stathmin binds free tubulin preventing its interaction with other proteins. Stathmin binding to tubulin is blocked by JNK-dependent phosphorylation, suggesting that JNK may promote menadione-induced cell death by decreasing stathmin-tubulin interactions, increasing tubulin binding to the mitochondrial voltage-dependent anion channel and thereby promoting loss of mitochondrial integrity and cell death. However, no increase in mitochondrial tubulin was detected by immunoblotting in menadione-treated cells with a knockdown of stathmin (data not shown), further indicating that the effect of stathmin in oxidant stress was unrelated to its ability to interact with free tubulin.

**Figure 8:**
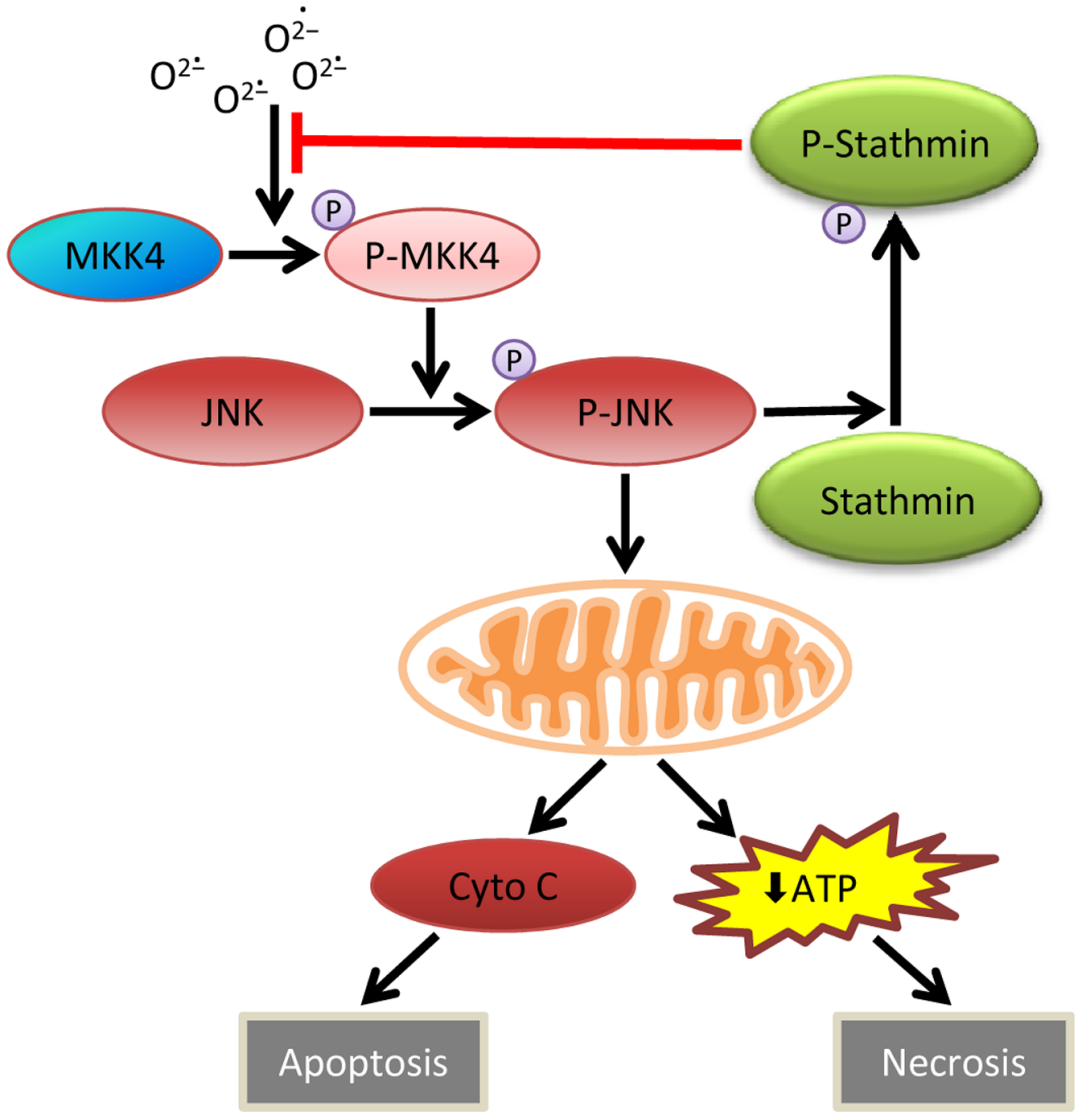
Working model of the regulation by stathmin of JNK-dependent hepatocyte death from oxidative stress. Increased superoxide generation triggers phosphorylation of MKK4 which then phosphorylates and activates JNK. If activated for a long enough period of time, JNK compromises mitochondrial integrity leading to cytochrome c (Cyt c) release and apoptosis or ATP depletion and necrosis. However, JNK also phosphorylates stathmin which acts through a negative feedback loop to suppress phosphorylation of MKK4 and its downstream substrate JNK to promote cell survival.

The present findings establish a new inhibitory feedback loop between stathmin and JNK/c-Jun signaling (Figure 8). JNK activation that occurred in response to oxidative stress led to stathmin phosphorylation and stathmin down regulated injurious JNK overactivation. The effect was not due to a direct stathmin-JNK interaction, but occurred upstream above the level of MKK4, and was oxidant stress dependent as it was blocked by catalase overexpression. Augmentation of mitochondrial energy stores by oleate supplementation blocked JNK overactivation in the absence of stathmin. This finding is consistent with the existence of a mitochondrial amplification loop whereby JNK-mediated mitochondrial damage generates increased reactive oxygen species that promote further JNK activation as recently described for endoplasmic stress-induced JNK overactivation in hepatocytes [52]. Oleate by maintaining mitochondrial energy homeostasis and integrity stops this amplification and blocks the prolongation of JNK activation.

The inhibitory effect of stathmin on JNK had the beneficial effect of promoting hepatocyte survival from injurious oxidant stress. Stathmin-JNK crosstalk may play a role in tumor biology as maintenance of mitochondrial integrity leading to cell survival during oxidant stress could explain in part the tumor-promoting effects of stathmin overexpression. JNK/c-Jun signaling also functions in tumor promotion [53], suggesting that JNK/c-Jun down regulation by stathmin may suppress tumorigenesis. However, recent evidence has suggested that JNK may also act as a tumor suppressor in hepatocellular carcinoma [54], and stathmin inhibition of JNK may reduce this tumor suppressor effect as another mechanism by which stathmin acts as a tumor promoter. Further investigations are needed to delineate the mutual interactions between stathmin and JNK that may regulate liver injury and tumorigenesis.
